# Financing assistive technology—Policy options on the path towards universal health coverage

**DOI:** 10.3389/fpubh.2026.1901297

**Published:** 2026-09-28

**Authors:** Jens Wilkens, Ranjavati Banerji, Satish Mishra

**Affiliations:** 1 Department of Clinical Sciences, Lund University, Malmö, Sweden; 2 ATscale, the Global Partnership for Assistive Technology, Geneva, Switzerland

**Keywords:** affordable access, assistive technology, cost-effectiveness, needs-based utilization, public financing, universal health coverage, user payment

## Abstract

Supporting functional ability in everyday life is essential for advancing toward universal health coverage (UHC). However, of the estimated 2.5 billion people in need of assistive technology (AT) globally, the vast majority do not have access. The main reason for this is financial barriers, particularly in low- and middle-income countries. This study aims to identify financing policy options for increasing access to affordable AT. The UHC concept, more specifically the three coverage dimensions—population, interventions (products and services), and costs, is used to descriptively analyze gaps in coverage related to affordability. The advantages and disadvantages of commonly practiced policy options, supported by global evidence, are explained and illustrated with country examples, resulting in structured guidance for both governmental and non-governmental policy stakeholders. When countries prioritize more strategically across the three coverage dimensions, financial resources can be targeted more effectively and have a greater impact. In addition, transparency about public financial commitments makes it easier for the non-government sector to contribute. Hence, public financing for AT is not only about raising more much-needed funds. There are also opportunities to increase access by designing publicly funded coverage transparently and guide the large non-governmental sector to be more effective in covering gaps.

## 1 Introduction

Globally, 2.5 billion people need one or more assistive products, and this estimate is projected to increase further with aging populations and the rising prevalence of non-communicable diseases (1). Assistive technology (AT) is an essential component in universal health coverage (UHC), providing substantial physical and mental health benefits (2). Effective AT helps individuals alleviate or mitigate functional disabilities in everyday life, increases participation in educational and professional life, and reduces the need for informal support from family members (Box 1). Accordingly, the return on investment in common assistive products has been shown to be 9:1, indicating that every dollar spent on AT yields 9-fold returns by creating large welfare gains (3). Despite its recognized benefits to an inclusive society and economic welfare, AT receives limited attention in public policy development and funding (4). In low- and middle-income countries (LMICs), only one in 10 people who need an assistive product actually access it (1).

**Box 1 This is assistive technology.**

AT is an umbrella term for the combination of assistive products and the systems and services needed to ensure safe assessment, distribution, and use of these products. An assistive product is any physical or digital device external to the human body, such as wheelchairs, hearing aids, prostheses, spectacles, or digital devices, whose primary purpose is to maintain or improve an individual’s functioning and independence and thereby promote their wellbeing. The prevalence of need and mode of provision for AT vary widely by type of assistive product. These products require diverse assessment methods and fitting skills. The products are provided not only through a wide range of health care services but also by education and social service providers. While some products are highly advanced and require specialists, others can be provided through task sharing by a range of professional categories, such as in community health centers (10).

The main challenge to accessing AT for people in need is affordability, with 40% of respondents globally indicating “cannot afford” as a barrier to access (1, 5). In all countries, user payments are more prevalent for AT than for healthcare generally. In LMICs, where social protection systems are generally weaker and people have lower individual purchasing power, the financial barriers to accessing AT are higher than in high-income countries (Figure 1), although for specific products financial barriers can be severe also there, such as hearing aids for older adults (44). In contrast to healthcare generally, AT needs are typically continuous and long-lasting, which increases financial vulnerability. For example, someone who permanently loses their ability to walk will need assistive products and associated services to support mobility for the rest of their life. These recurrent expenses exacerbate the financial burden of out-of-pocket payments (OOP). In addition to financial barriers and consequences for the individual, the high prevalence of user payments also creates inequities and inefficient use of societal resources when AT utilization is based on ability-to-pay rather than need.

**Figure 1:**
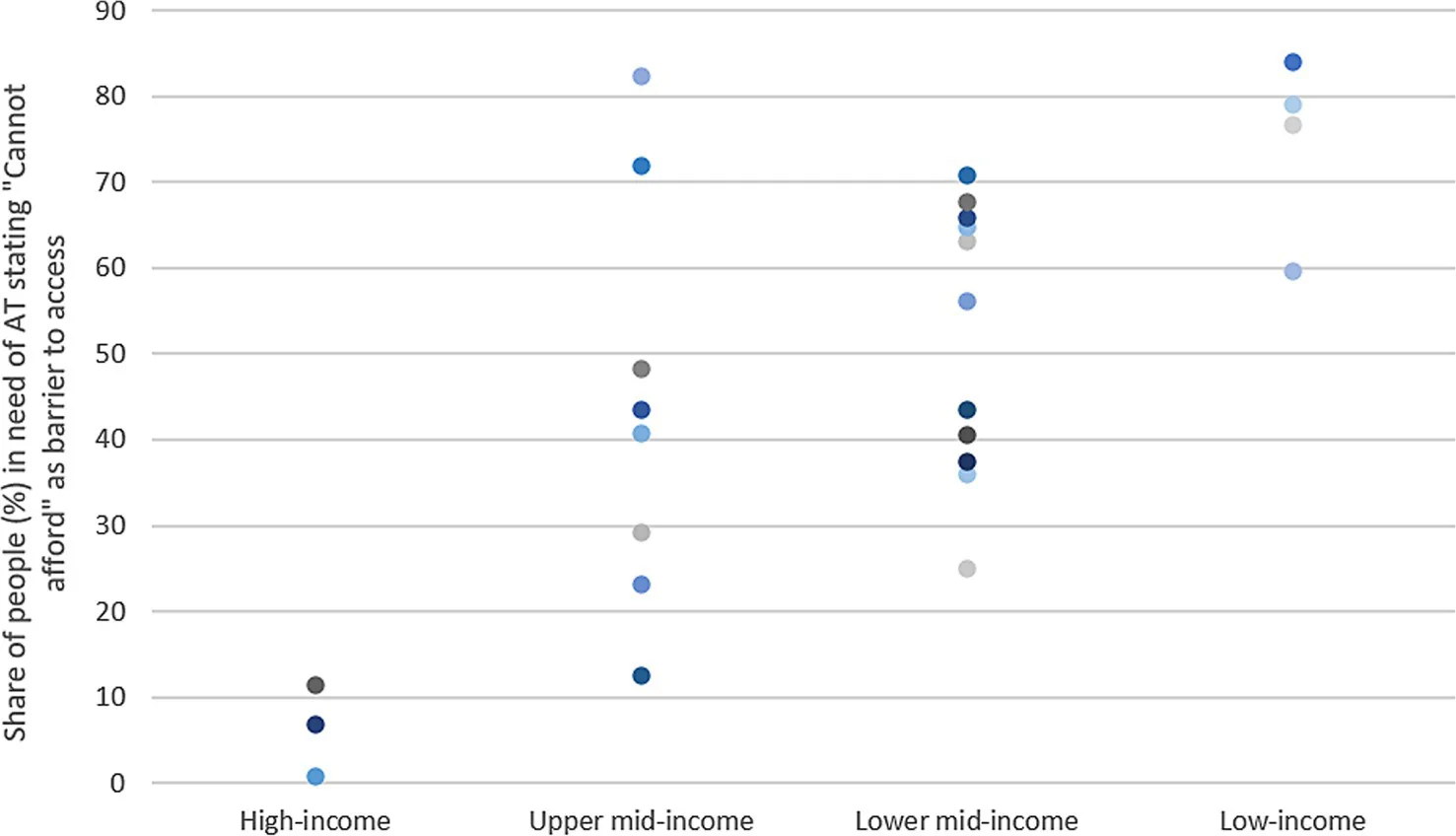
Affordability is a major barrier to accessing AT in many LMICs. Source: The rapid assistive technology assessment (rATA, www.who.int/tools/ata-toolkit/rata) has survey data on this indicator from 26 countries, each representing a dot in the figure. Country income groups by World Bank classification. High-income: Sweden, Italy, Poland; Upper mid-income: People's Republic of China, Maldives, Jordan, Dominican Republic, Iraq, Georgia, Azerbaijan, Guatemala; Lower mid-income: Mongolia, Ukraine, Iran, Tajikistan, Myanmar, Nepal, Kenya, Djibouti, Senegal, Indonesia, Pakistan; Low-income: Burkina Faso, Malawi, Togo, Liberia.

### 1.1 Aim and method

To advance toward UHC, meaning that all people can access the health services they need without suffering financial hardship, public funds are absolutely essential (6). However, all countries have constrained public budgets, and many experience a tightening fiscal environment (7). Increasing technical and medical opportunities also contribute to ever difficult priorities of public financial resources. When priorities are not strategic and systematic, based on data-driven knowledge about needs in the population, there is a risk of implicit rationing, i.e., an unspoken and indirect limitation of access or quality because of unmanaged scarce resources (8). The AT sector is no exception to this, as evidenced by vulnerable people having to pay directly out of pocket for AT. This study aims to specify financing policy options for increasing access to affordable AT. While the starting point is financing a public commitment, in support of sustainability and resilience, the financing policy options in this study can contribute to increasing the scope and effectiveness of the important contributions made by non-governmental stakeholders in the AT sector.

Access to affordable AT is operationalized using the three UHC dimensions population coverage, intervention coverage (products and services), and cost coverage (9). The three dimensions are used to descriptively analyze gaps in AT coverage and define options in financing policy for addressing these gaps. Originating from the health sector, the UHC framing is well suited to describe coverage in all related sub-sectors, including those in which the interventions are dependent on other sectors such as education and social policy. Examples from countries globally, identified through targeted searches of peer-reviewed and gray literature, e.g., including reports from international organizations, are used for the illustration of policy options. The examples are selected purposively to inform the conceptual arguments developed in the study, drawn from different geographical and health system contexts, and should be interpreted as illustrative rather than evidential. The examples were selected to reflect variation in the income level, region, and AT coverage. Table 1 provides an oversight of the options.

**Table 1:** Summary of policy options framed in three UHC dimensions.

UHC coverage dimensions	Policy options	Strengths	Weaknesses
Population coverage	Formal disability status	Explicit and transparent	Fails to include large groups in need
	Functional ability assessment	Comprehensively targets need	Costly Capacity demanding
	Socio-economic discrimination	Prioritizes vulnerable groups	Dependent on information systems
Product and service coverage	Cost/budget impact	Cost-containment/budget discipline Simple (low-cost)	Does not relate to need and utility
	Effectiveness or utility	Relates to desirable and relevant outcomes	Does not relate to the intervention cost
	Cost-effectiveness	Enables the allocation of resources relative to outcome	Standard outcome matrixes for AT do not exist
Cost coverage	User charges	Allows budget to cover more people or interventions	Creates financial barriers Does not prioritize among interventions Risks a regressive distribution
	Percentage share	Controls budget	Low financial protection
	Fixed amount	Transparent High financial protection	Higher budget expense
	Cap or deductible	Lowers financial barriers	Dependent on information systems Higher budget expense
	Deferred payment	Lowers financial barriers	No sharing of risk and funding burden

All policy options presented the need to balance different specific objectives, beyond the overall UHC ambition, and thereby come with both advantages and disadvantages. Deciding whether public resources should be spent on reading glasses is a case in point. Reading glasses have proven high impact relative to their low cost, particularly if the effect measurement are education or labor income (46), hence they can have high priority from a cost-effectiveness perspective. However, they are also relatively low-cost for the individual user, i.e., the financial burden on households obtaining reading glasses at their own expense may be low if the retail market works well. This may give them a different priority from the perspectives of equity and financial protection.

## 2 The UHC framework in three dimensions

The UHC concept is made concrete in the sustainable development goals (SDGs) through the SDG 3.8 target, which incorporates service coverage (3.8.1) and financial protection (3.8.2) (11). A commonly applied framework for more comprehensive and policy-oriented description and assessment of UHC is to describe UHC in the three coverage dimensions—population, interventions (products and services), and costs. The cost dimension describes the share of the cost of a given intervention that is covered by public funds and how much is left for the user to pay in order to be able to obtain the interventions. The three dimensions are graphically illustrated in Figure 2. This framing is effective in assessing public resource priorities and benefits packages in health (9), as each dimension can be defined and described for any country’s health system, or for a specific health or social service area separately.

**Figure 2:**
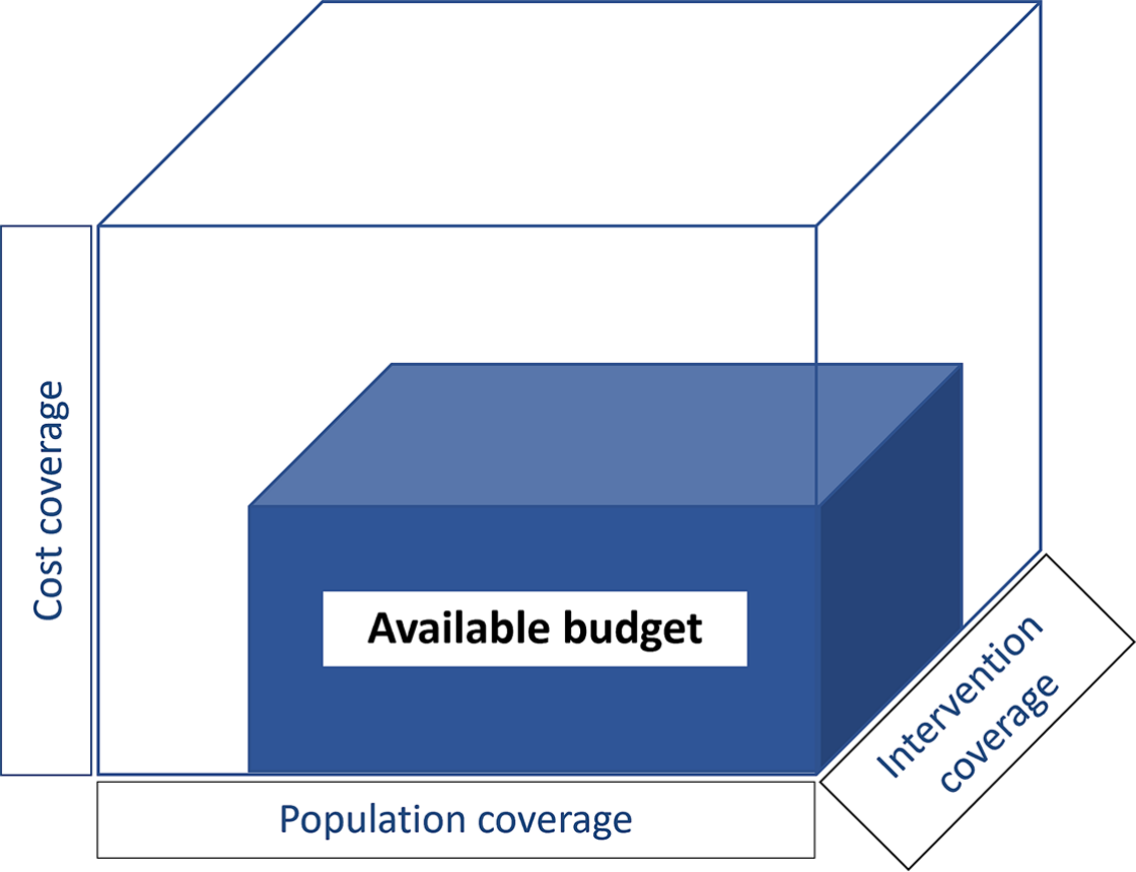
Universal health coverage in three dimensions. *Source*: Adapted from World Health Report 2010: Health systems financing—the path to universal coverage.

Applied to AT, it illustrates the priority choices that any country has along the dimensions: (a) who in the population is entitled to publicly funded AT, (b) which assistive products and services within AT are to be publicly funded, and (c) to what extent users are required to cover the costs along these other two dimensions (as any intervention for any beneficiary can be fully or partly funded by the public purse). Accordingly, the total public funding commitment is represented by how each dimension contributes to coverage and by the specific priority options made along these dimensions. Note that there are important aspects not covered by these three dimensions. Most notably, quality is absent, sometimes called the fourth coverage dimension (12). Quality and safety can certainly be affected by resource levels and financing mechanisms, but they fall outside the scope of this study.

Unfunded ambitions about coverage in one or more dimension are promises in benefit entitlements that the budget cannot carry and does not help the system to provide affordable AT based on needs. Overpromising risks leading to undesirable effects such as informal payments, deteriorating quality, waiting times caused by stock outs and shortages of human resources. Prioritizing by explicitly and transparently defining coverage can support the purposeful use of financial resources.

## 3 Shaping the UHC box for AT

### 3.1 Population coverage

In most countries, the legislation for health care and social services regulates AT eligibility. Countries with residency-based entitlements funded by general government budgets (national or local) rely on more general descriptions of rights and needs, while countries with (more or less compulsory) contributory-based entitlements have a tradition of more detailed specifications of both beneficiaries and interventions (13). Many LMICs, where contributory health insurance has been introduced in recent years, struggle to reach full population coverage, largely because of challenges in covering groups outside the formal labor market (14).

#### 3.1.1 Population coverage options—priority along need and social vulnerability

Although the goal “universal” indicates that everybody in society is entitled, all countries discriminate social protection benefits to some extent through various targeting policies (15). In the UHC concept, *need* is an essential aspect, and in systems with universal access to health services, this is often medically defined, such as care provided based on certain indications. A common approach to defining population coverage in AT is formal disability status (16, 17). The formal disability status is an explicit prioritization mechanism that transparently guides users and providers but carries a set of shortcomings in allocating financial resources. Because population coverage of AT is not simply about disability, it fails to include large groups of people, such as those who have temporary impairments, impairments related to aging, or those who simply cannot navigate the disability registration process. The approach is sensitive to the definition of disability and how well it captures functional ability, for example, complex cases with a cumulative impact of multiple conditions. It can also be an administratively costly approach for both users and the provision system, as approval of a disability status often comes in addition to the clinical assessment (18).

Using the need for support for functional ability as a criterion rather than disability has the potential to allocate AT resources for a broader set of needs, but is arguably more costly. Classifications and validated standardized assessment scales for individual functional status are global public goods but these assessments are capacity and resource demanding. Decisions about an individual’s eligibility typically involves both assessment by health professionals such as occupational- or physiotherapists and social services or other funding bodies (19).

Prioritizing the population based on socioeconomic or demographic discrimination, targeting social protection systems toward the most vulnerable can be effective in reducing financial barriers to access, as poor people are furthest away from utilization and more prone to financial hardship. The choice of specific sociodemographic criteria to capture financial vulnerability depends not only on available administration and information systems but also represent differences in priorities, as means-testing of the individual by wealth or income will have a different impact than simpler and administratively low-cost criteria, such as age or residency. In addition, this approach can be administratively costly, both for users and the responsible public administration. It requires not only a means-testing system already established for other purposes, but also an information exchange between the social security and AT provision system.

Many countries combine different approaches to needs- and means-testing to prioritize resources. In Indonesia, persons with disabilities who are in the two lowest income quintiles have access to fully subsidized health insurance, which covers a defined set of assistive products. India combines functioning status and means-testing, where a degree of assessed disability greater than 40% gives eligibility to different levels of assistive product subsidy depending on whether the household income falls below defined thresholds (16). To be effective, these models require capacity on the clinical level to assess the functioning status, administrative capacity to assess household wealth, and information exchange between multiple public agencies. In the absence of these capacities, the effectiveness in prioritization decreases.

### 3.2 Assistive product and service coverage

AT is often not included in UHC programs and concrete tools such as benefits packages, in particular not in LMICs, where curative and acute medicine are traditionally prioritized over rehabilitation (4), although this is slowly changing with the increasing recognition of the sector. The specific products and services to include in benefit packages when funding is available requires concretization of interventions, as arbitrary choices and implicit priorities will otherwise prevail. A concrete tool, increasingly implemented in countries, is the priority assistive products list (APL) introduced by WHO. This global list identifies essential assistive products, which any country can develop into its own national priority list based on local needs and resources. Similar to the lists for essential medicines (20) and diagnostic services (21) by WHO, it provides an important basis for identifying essential assistive products that need to be available. Notably the global APL serves several purposes, such as definition and regulation of quality standards and technical specifications, price regulation, and procurement specification (22).

#### 3.2.1 Assistive product and service coverage options—priority along cost and effect

With constrained budgets, a policy must establish priorities about which assistive products and services the public system shall fund. Most often, the decision-making process is primarily guided by the budget impact, i.e., the intervention cost multiplied with an estimate of volume such as the need for the intervention. However, without evidence on the effectiveness of any product or service choice, budget impact on its own is not enough to decide about using public funds.

Measurements of effectiveness are scarce in AT, and the methods to assess it, including the level of effectiveness of different alternatives, vary considerably across countries. Validated AT outcome measures for comparing effectiveness across both different AT domains and products are simply not available yet (23, 24). The APL is a useful tool and when applied at the country level, national context adjustments can be done, for example, to ensure budget impact is manageable. However, in economic evaluation of interventions, health technology assessment and benefit design, the question is not whether an intervention cost is low enough to be affordable for a budget or whether it is effective in providing functional ability, as these criteria can point in different directions. The decisive criterion is the ratio between the two, the cost-effectiveness or cost-utility (25). The 2018 World Health Assembly resolution *Improving access to assistive technology* explicitly urges member states to develop a national priority APL that is “affordable and cost-effective” (47). Standard economic evaluation methods in healthcare use quality-adjusted life years. This is difficult for comparing AT effects, as the value is in function rather than in health outcomes, and standards for assessing AT from this perspective (cost relative to effectiveness or utility) have not yet emerged in literature or practice (26).

Initiated and developed for advocacy and engagement to raise awareness of the vast societal value of allocating more resources to AT, studies on return on investment show high returns for all categories of AT, however with differences between them (3). The investment case and cost-effectiveness approaches stem from the same idea to relate input to outcome. However, return-on-investment studies are insufficient for guiding specific priority-setting decisions as these require more specific cost-effectiveness and equity to guide decisions regarding which assistive technologies, services, or delivery approaches should be prioritized in specific settings. This type of evidence is currently scarce.

### 3.3 Cost coverage

To any specification of who is entitled to which interventions, there is a choice about how the users are required to cover a large share of the cost by themselves. User payments come with severe challenges to access and are detrimental to efficiency as utilization of resources becomes governed by the ability to pay rather than need. However, they are not realistic to eliminate from the AT sector in most LMICs. Rather, financing policy needs to mitigate negative effects to the extent possible.

#### 3.3.1 Cost coverage options—priority along budget and user affordability

Many countries apply co-payments to partially cover interventions that are publicly funded, often justified from the budget perspective, as it will cover more products or people when costs are shared with the user. The main immediate risk is the financial hurdle for people with a low ability to pay. However, it does not help users who can afford to pay themselves to distinguish between high- and low-value AT, which can also lead to the inefficient use of resources (27). In addition, spreading public resources thin by applying user fees across a broad range of products to “afford more” does not prioritize the scarce funds among interventions. Furthermore, the risks of partially funding interventions that public funds are directed toward relatively advantaged affluent people, as only those who can afford the remaining copayment will utilize the product or service.

The cost coverage structure can be designed to mitigate some of these risks. A public subsidy up to a reference price for each product, or a yearly cap (ceiling amount) for the individual, can be applied. Countries with advanced administrative infrastructure and information systems can combine dimensions of cost for the population coverage to target vulnerable groups specifically, such as full cost-coverage for severe functional limitation (Portugal applies a threshold of 60% or more functioning incapacity) or capped copayments for defined assistive products (Netherlands limits co-payments to 385 Euros per year) (28). However, these mechanisms come with information needs and transaction costs. For countries with weak information systems, the risk of high administrative costs outweigh the benefits of cost-sharing arrangements (29). Generally, fixed flat-rate copayments are easier to make transparent and protect users from high-cost AT better than cost-sharing based on a proportion (percentage) of the cost. Fixed copayments may also give incentives to the purchasing agency to keep prices down, as the individual user meets the same cost.

Deferred payment models such as consumer loans and microcredit arrangements are also techniques to decrease the funding burden even when the user has to cover costs themselves. Credit schemes provided by product vendors, government-supported programs, or separate credit institutes are common in AT in specific countries (30) and can avoid unmet needs and alleviate financial hardship. Digital payment solutions may also increase efficiency in these mechanisms and make them more accessible (31). However, while these mechanisms can lower the immediate financial barrier for the individual and enable access to a product at a specific point in time, funding is still sourced from the individual user and only redistributed over time. They do not use the benefits from pre-paid pooled resources that can share the financial risk and burden across the ability-to-pay and need.

The alternative to partially cover costs between the public funding scheme and the user is to be more explicit about what is fully funded by the government and what should be paid by the AT user, i.e., strictly prioritizing across the products and services dimension. Leaving the full cost to the individual in need can be an explicit policy choice but is arguably more often a residual effect of not making a choice. But just as it is possible to specify the essential AT that should be funded, priority setting can also include criteria for when public funds should not be used (32). AT interventions that are low cost meet needs with relatively short time frame, address relatively lower levels of functioning difficulty, and carry less risk that the ability-to-pay is decisive for access. Especially if exemptions for vulnerable users are possible (when information systems and regulations are advanced). Instead of partly funding these AT interventions, scarce budget funds can be directed toward the most cost-effective AT, publicly funded at zero co-payment. This explicit separation builds on the evidence that the responsible public agency has the capacity to assess a full range of AT by multiple criteria, so that the selected fully funded AT interventions are well targeted.

## 4 Expanding the UHC dimensions by increasing funding for AT

Increasing access to affordable AT can also be achieved by increasing the size of “the box,” that is allocating more public funds to the sector. Budgetary prioritization within the general government budget is the starting point for how much is available to AT. Government budget practices for AT are highly heterogeneous across countries, and the actual priority given to AT in government funding is difficult to assess because of the often multiple responsibilities across line ministries and levels of administration.

In the health sector, there is an increasing practice of raising taxes to disincentivize unhealthy consumption like tobacco or alcohol, with the additional policy option to earmark these funds for a specific health-related purpose. Instead of reallocating existing scarce public funds, raising new revenue this way can serve dual purposes by nudging behavior and generating revenue (33). Careful design of such policies is needed to avoid potentially negative effects. First, different consumption taxes have different distributions of contributions across the population, with a risk that low-income people become the funding source. Road and aviation traffic fees are examples with relatively more high-income contribution effects. Second, using these funds for an overly narrow pre-defined specification of budget purpose or end use, often called earmarking, can cause inequities. For example, if traffic fees are earmarked for AT needs caused specifically by traffic injury rather than serving the same AT need regardless of cause, individual access to AT becomes inequitable. In Canada and South Africa, a fuel levy is used to rehabilitate people injured in road accidents, whereas an alternative is to fund the same intervention regardless of the cause of injury. Third, earmarking resources for a specific purpose already at the source can cause inefficiencies by impeding flexibility when newly identified or urgent needs are identified, cause unnecessary fragmentation, or even become an argument for not allocating general budget resources to the same area. Finally, raising funds in this way requires consideration of the fact that, when undesired consumption declines, the fund-raising ability decreases. The collective body of evidence suggests that taxes on undesired consumption can be an effective complement in raising revenue for health purposes but should primarily not be designed for an overly narrow purpose (21).

## 5 Pooling funds with common purpose to increase UHC

Funds allocated to AT are characterized by high fragmentation, even more so than the health and social sectors generally. Responsibility is often spread along a continuum of services. For example, the need for an assistive product supporting mobility may be identified in school, eligibility for support may be dependent on means-testing by another authority, and fitting and training for the actual product may be provided in healthcare. Of the 70 surveyed countries in 2021, half of the countries had AT funds allocated across three or more ministries (1), in addition to often multiple levels of local and regional administrations. An Australian study identified 88 government funders, administering 109 funding schemes for AT (48). Non-governmental organizations, in many contexts that are crucial for meeting access gaps, also add to the fragmentation, particularly in LMICs.

Aligning different funding streams in AT can enable resource distribution by need, scale procurement and grow purchasing power for lower prices, and support tasks such as consolidating needs assessments and develop guidelines. In the pharmaceutical sector, pooling of funds has been used for many years to leverage global purchasing power for the benefit of both price and quality (43) and for nationally centralized purchasing (45). The same principles are applicable for AT financing, both globally and in national contexts. With multiple revenue streams and administrative sector responsibilities, including development assistance, civil society, and small enterprise contributions, pooling resources across these different entities can help purchasers leverage their market power. Because NGOs are important stakeholders in the AT ecosystem, pooling is not only about government funds. Charity funding is small relative to total AT funding but an important part for specific interventions and population groups (34). A case in point is Consolidating Logistics for Assistive Technology Supply and Provision (CLASP), a social enterprise that consolidates demand from buyers of mobility devices such as wheelchairs, postural support devices, and walking aids. A pooled procurement mechanism enables multiple buyers, including government agencies, NGOs, and charities, to combine their individually relatively low purchasing power to leverage larger procurement arrangements and increase the respective organization’s range of products (35).

## 6 Thinking outside the UHC box—shifting coverage focus from product access to utilization and impact

Attaining UHC is not the final goal; rather, it is a means to achieve equitable improvements in outcomes in the case of AT by achieving the highest possible functional ability for all. Hence, UHC must not be limited to finding more resources and increasing intervention and cost coverage (36). Traditionally, public funding of AT has been about paying for products and required associated services. If funding is directed to make products available for use rather than disbursing them, providers have more interest in extending the product’s lifecycle. If also associated services are part of the funding agreement, the integration of products with services is supported. In Tajikistan, 46% of respondents stated that there is “rarely or never” a place to go for repair or maintenance of the assistive product, a challenge currently met by integrating a set of essential AT into primary health care services and its associated funding model (37). A product example is hearing aids, which can be expensive for the individual user. However, the lifecycle cost also includes fitting, training, repairs, and replacements of batteries and ear domes or ear molds, which illustrates how interdependent service provision and financing are in ensuring that the product be effective (38).

In Norway, lending equipment is part of the service delivery model (19), but globally such models are still rare (39). Moreover, when users fund the products themselves, similar models can lower cost barriers by reducing the up-front expenses when the user pays for renting instead of obtaining the product. The model can include the assessment and product fitting prior to purchase and cost implications of maintenance and repairs of products after purchase.

The next step is to move focus from utilization to what is actually desired; long-term impacts, such as increased functional ability, or even increased utility for the individual, such as labor participation (33). Social, or health, impact bonds, used across health, education, and employment, are financing mechanisms where the purchaser reimburses the provider if predefined outcomes are achieved, with the aim to incentivize the measurable impact. These models are best suited when the provider is responsible for a larger part of the AT continuum than only products or single services. Areas in AT with potential for impact bonds, similar to how it is used in the social sector, include impacts on labor participation or children’s schooling by means of AT.

## 7 Conclusion

To meet the needs of affordable AT, more public funding for AT is required. However, the sector can also make better use of currently available resources. By applying the UHC framework, this study meets a gap in the global literature about how to develop health financing principles and practices to the field of AT. As the AT market in each country looks different, and the public financing systems vary, the specific context in each country is decisive for the choice and specific design of any of the policy options discussed in the study.

Much-needed efforts to strengthen public policy in the sector indicates that financing policy is both about the public spending as such and about enhancing the use of private financial and non-financial resources. The large group of non-government stakeholders in the AT ecosystem can become more effective in their missions when the public commitment is clear about who in the population is eligible and which products are funded, as uncertainty and fragmentation currently cause inefficiency and lost opportunities among suppliers, charities, and AT professionals (40). Hence, clarity about which AT is publicly funded and which policies are conducive to the effective use of private efforts can support moving toward UHC.

Finally, an important enabler for shaping the policies assessed in this study is to close the large gaps in data availability about need and utilization. Without knowledge about who needs what, and how their need is currently met, it is difficult for countries to improve financial allocation as sparse and disparate data hampers informed policies. As described above, performance monitoring in function and disability is traditionally weak in the sector. Without a robust value matrix for comparing effect and impact of AT, it is challenging to make priorities (24, 41, 42). In many countries, there are opportunities in integrating AT services and product utilization data into the health information systems, which could increase data availability.
